# Health Literature

**Published:** 1871-06

**Authors:** 


### HEALTH LITERATURE.

FOR a number of years HALL'S JOURNAL OF HEALTH was the only magazine devoted exclusively to the instruction of the people as to the importance and the best method of preserving the health by proper habits of living. But a taste for such reading has been cultivated, and has spread to such an extent as to warrant similar publications in different parts of the country, all of which are doing good service, and there is room for others, without interference with this JOURNAL; all help to extend the area for reading about health, and this redounds to the advantage of each.

"Good Health," Boston, $2 a year, is devoted to the improvement of human health and lengthening human life; it is edited with ability, and deserves a wide patronage.

"Health and Home," 805 Broadway, New York, De Puy

Brothers, publishers, monthly, $1.50 a year. The first number appeared in March, and every page has most valuable and reliable instruction in health matters, and deserves a large circulation.

"Herald of Health," 15 Laight Street, New York, $2 a year, is the exponent of water cure ; every number has a large amount of instructive reading matter about health and disease. Valuable articles often appear in relation to the management of children. Mrs. Gleason gives excellent advice to parents, in the March number, about the education of daughters, which has been extensively copied in family newspapers.

"The Phrenological Journal," merged with "Life Illustrated," always abounds in lessons of temperance, virtue, morality; explaining and enforcing the laws of life ; $3 a year, 389 Broadway, New York, Samuel R. Wells editor and publisher. There is room, abundant room for all of us. But it is a shame, and a disgrace to the whole American people, that all these publications, this Journal included, have not one fourth the circulation claimed by some weekly newspapers, filled with nothing in the world but sickening love stories, tales of crime, and vice, and murder, and bloodshed, with coarse wood engravings a mile square, more or less ; to say nothing of columns of stale wit, disgusting, profane, and indecent anecdotes, read by tens of thousands of boys, apprentices, shop-girls, and the like, corrupting their minds, pandering to their passions, and unfitting them for the performance of any of life's duties. This Journal has increased its total circulation fivefold since the first of January, — not a single deadhead ; and we wish that the other similar publications will be able to say the same before the year expires ; they deserve it, for they are engaged in a good work.

